# SUMOylation-dependent degradation of nucleocapsid is responsible for *Pestivirus* uncoating

**DOI:** 10.1128/jvi.01648-25

**Published:** 2025-11-25

**Authors:** Lin-Ke Zou, Ji-shan Bai, Rui-cong Sun, Han-fei Yang, Ming-yue Wan, Bing-qian Zhao, Bo-tao Sun, Jin-xia Chen, Jing Chen, Yan Cheng, Bin Zhou

**Affiliations:** 1 MOE Joint International Research Laboratory of Animal Health and Food Safety, College of Veterinary Medicine, Nanjing Agricultural University 261674, Nanjing, China; 2 Key Lab of JinHua Pig Genetic Breeding and Improvement, Jinhua University of Vocational Technology 686684, Jinhua, China; 3 College of Veterinary Medicine, Northeast Agricultural University 12430, Harbin, China; 4 Northeast Science Observation Station for Animal Pathogen Biology, Ministry of Agriculture and Rural Affairs, Harbin, China; Emory University School of Medicine, Atlanta, Georgia, USA

**Keywords:** *Pestivirus*, SUMOylation, uncoating, Core, proteasome, valosin-containing protein (VCP/p97)

## Abstract

Classical swine fever virus (CSFV), a highly virulent member of the *Pestivirus* genus, is one of the most significant pathogens within this group. Although uncoating is a prerequisite for productive infection, the molecular determinants orchestrating this process remain obscure. The Core protein, functioning as the viral nucleocapsid, plays a pivotal role in the uncoating cascade. This study delineates a SUMOylation-dependent, ubiquitin-independent proteolytic mechanism essential for CSFV uncoating. The valosin-containing protein (VCP/p97) preferentially associates with SUMO1-modified Core, directing it toward degradation via the 26S proteasome, specifically through engagement with PSMB2 and PSMD2 subunits. Site-directed mutagenesis of the SUMOylation motif abolishes VCP-mediated degradation, substantiating its functional indispensability. Fluorescent tracking of CSFV virions using molecular beacon and quantum dot labeling further reveals that VCP governs the endosomal trafficking of viral particles from early to late endosomes—an essential step for capsid disassembly and genome release. Moreover, VCP operates in concert with NPL4 and UFD1, enabling the translocation of SUMOylated Core toward the proteasomal machinery. Collectively, these findings uncover a previously uncharacterized SUMO1-VCP-PSMB2/PSMD2 axis that couples intracellular trafficking with proteasomal disassembly of the CSFV Core, providing mechanistic insights into *Pestivirus* uncoating and nominating host factors as promising antiviral targets.

**IMPORTANCE:**

The fusion of the viral membrane and genome release are hallmark events of enveloped virus infections. However, the related dynamic mechanisms of most viruses remain poorly understood. Here, we demonstrate that VCP directly interacts with the CSFV core protein, and the core protein undergoes proteasomal degradation mediated by the PSMD2 and PSMB2 subunits, with VCP acting as a critical mediator. Surprisingly, this degradation process is independent of ubiquitination but exhibits a strong correlation with the SUMOylation of the nucleocapsid protein. In addition, we found that CSFV genome uncoating occurred in late endosomes, a process regulated by the host VCP. Depletion of VCP prevents viral trafficking to late endosomes and thereby disrupts uncoating efficiency. This is the first evidence implicating SUMOylation in viral uncoating. Deciphering the molecular intricacies governing viral uncoating is pivotal for propelling the development of broad-spectrum antiviral therapeutics aimed at the *Pestivirus* genus.

## INTRODUCTION

Classical swine fever virus (CSFV), a prominent member of the *Pestivirus* genus within the *Flaviviridae* family, is the causative agent of classical swine fever (CSF) in both domestic and wild pigs. CSFV establishes persistent infections in swine, often leading to chronic disease (1). Even in nations where CSF was previously eradicated, the disease has reemerged (2, 3). CSFV is a positive-sense, single-stranded RNA virus about 12.3 kb in length, consisting of an open reading frame (ORF) surrounded by two untranslated regions (UTRs) (4). This genome encodes a polyprotein that is cleaved into four structural proteins (capsid protein Core, envelope glycoproteins Erns, E1, and E2) and eight non-structural proteins (Npro, p7, NS2, NS3, NS4A, NS4B, NS5A, and NS5B) (5, 6). Over the past decades, substantial insights have been gained into the molecular mechanisms governing viral genome replication (7–10). Nevertheless, the processes involved in viral genome assembly and uncoating remain poorly understood. As the virus traverses the endosomal pathway, conformational changes in the envelope glycoproteins mediate membrane fusion, enabling the release of the viral nucleocapsid into the cytoplasm (11). Nevertheless, the precise mechanism by which the viral genome is subsequently released to initiate protein synthesis during infection remains unclear in the CSFV life cycle.

The CSFV Core protein has been considered a structural component essential for nucleocapsid formation and RNA packaging. The CSFV core protein is a small protein rich in basic amino acids located at the N-terminus of the viral polyprotein (after the Npro). It requires processing at the N and C termini in order to be released from the polyprotein. These cleavages are performed by the virus-encoded autoprotease Npro and the host cellular SP between Cys_168_/Ser_169_ and Ala_267_/Asp_268_, respectively. The final mature CSFV core protein is about 80–90 aa long (12). Similar to other members of the *Pestivirus* genus, the CSFV capsid protein is a small, highly basic polypeptide with strong affinity but low specificity for binding to the viral genome (13). It also interacts with viral non-structural proteins and host cell proteins. These interactions play a crucial role in modulating viral virulence, vitality, and replication. Through the application of yeast two-hybrid, several host proteins interacting with the CSFV Core protein have been identified, further substantiating its involvement in viral pathogenicity (14–16). Despite these advancements, studies on the CSFV Core have made limited progress, particularly concerning its connection with CSFV uncoating.

VCP, a member of the AAA+ family of ATPase proteins, mediates a broad spectrum of cellular functions (17). It functions as a segregase protein that directly recognizes ubiquitin signals on target proteins with the help of adaptor proteins (18). Utilizing ATP hydrolysis, VCP unfolds ubiquitinated proteins and dislodges them from larger molecular complexes (19, 20). Beyond its canonical role in recognizing ubiquitinated substrates, accumulating evidence suggests that VCP also engages SUMOylated proteins, particularly in the context of SUMO–ubiquitin hybrid signals generated by SUMO-targeted ubiquitin ligases such as TOPORS and RNF4 (21). These combinatorial modifications facilitate substrate unfolding and proteasomal targeting by the UFD1/NPL4/VCP complex, thereby mitigating the accumulation of misfolded or potentially cytotoxic protein aggregates. Co-factors such as CLIP-cohibin complex further modulate substrate specificity, collectively positioning VCP as a key integrator of SUMO- and ubiquitin-mediated proteostasis pathways (22). VCP has been involved in numerous viral contexts, particularly those within the *Flaviviridae* family; these positive-sense RNA viruses exploit VCP during early stages of infection. Our previous studies have demonstrated that VCP is a critical host factor essential for CSFV replication (23). Specifically, suppressing VCP expression or enzymatic activity disrupted the transport of virions from early endosomes to lysosomes. Similarly, studies utilizing a replication-defective reporter virus system demonstrated that DBeQ and NMS-873, two small-molecule inhibitors of VCP, effectively impeded the uncoating of Yellow Fever virus (YFV) (24). Likewise, DBeQ and NMS-873 sensitivities were observed in Dengue virus (DENV) (25) and Zika virus (ZIKV) (26), indicating that VCP may be universally required across *Flaviviruses* (27, 28). Furthermore, experiments using small interfering RNAs (siRNAs) targeting VCP inhibited early stage of West Nile virus (WNV) infection (29). Recent findings with Japanese encephalitis virus (JEV) indicate that the VCP inhibitor successfully blocked viral replication in both tissue culture and a mouse model (30). Given that VCP impacts multiple cellular processes, additional studies have corroborated the requirement of VCP for the replication of diverse viruses (31–38). Considering its pivotal role in viral replication, VCP emerges as a highly promising target for the development of antiviral therapeutics, with broad implications for treating a variety of viral infections.

SUMOylation is a ubiquitination-like post-translational modification that plays a pivotal role in regulating protein functions. Recent studies have shown that proteins from both RNA and DNA virus families can be modified by SUMO conjugation, and this modification appears to be crucial for the functionality of viral proteins (39–41). SUMOylation has widespread effects on various cellular processes, including transcriptional regulation, apoptosis, stress responses, and cell cycle control, making it an attractive target for viral dysregulation. The capsid structural protein of human papillomaviruses (HPV) is also SUMOylated, and this modification enhances their stability (42). The DENV envelope protein binds to Ubc9, and overexpression of Ubc9 leads to reduced plaque formation (43).

In this study, we identified that the VCP played a pivotal role in CSFV uncoating and CSFV Core protein degradation. Mechanistically, VCP plays a pivotal role in the translocation of Core from the early endosome to the late endosome. Moreover, CSFV Core protein undergoes SUMOylation in a SUMO1-dependent manner, with VCP interacting with the SUMOylated Core to mediate its subsequent degradation. The SUMOylated Core is then degraded through the ubiquitin-independent PSMB2 and PSMD2 26S proteasome system. Collectively, our findings highlight VCP as a key host factor involved in CSFV uncoating, presenting significant potential for the development of broad-spectrum antiviral strategies targeting *Flaviviridae*.

## RESULTS

### VCP interacts with Core and degrades it

**Fig 1:**
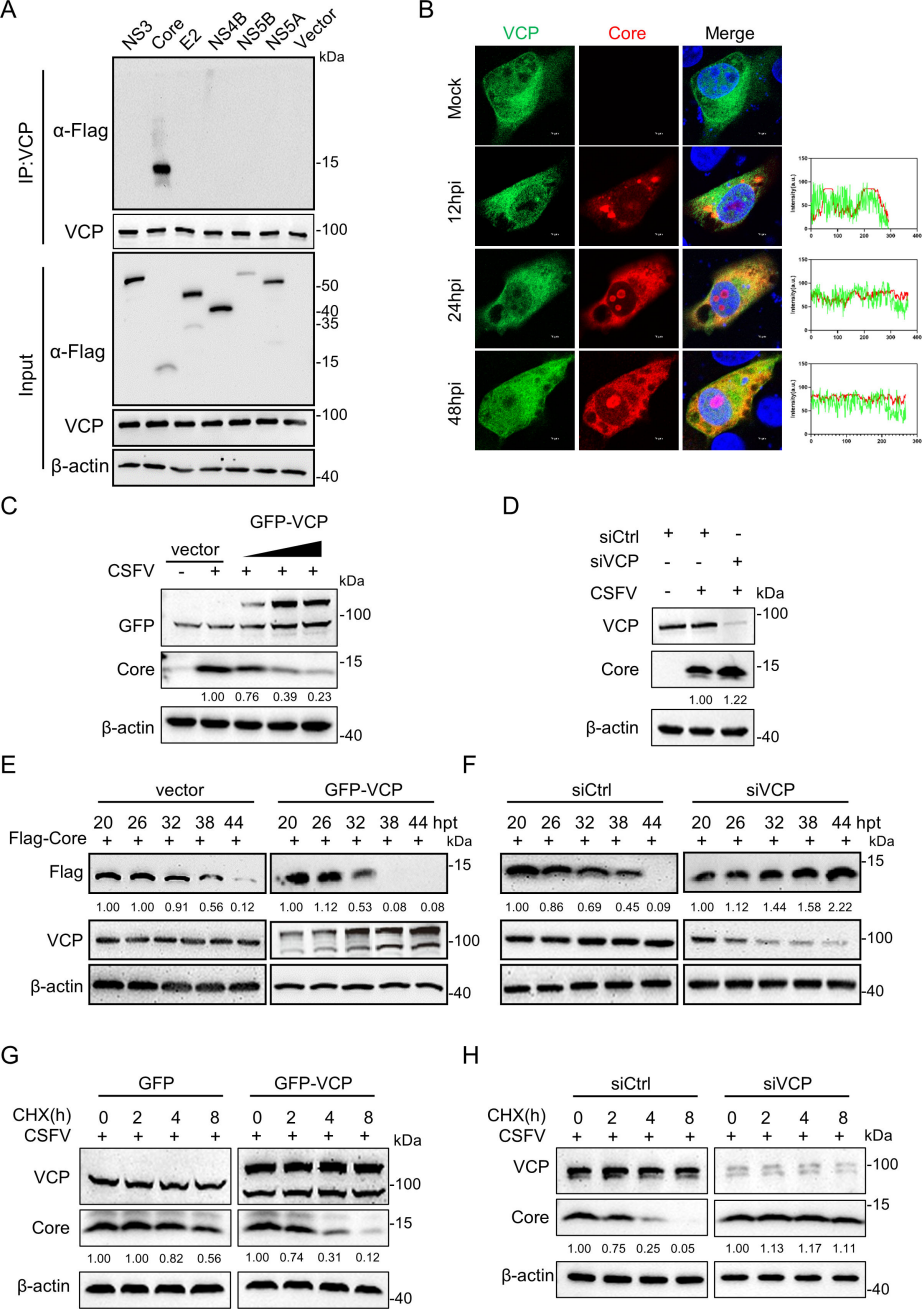
VCP interacts with CSFV Core and degrades it. (**A**) PK-15 cells transfected with pFlag-NS3, -Core, -E2, -NS4B, -NS5B, -NS5A, or vector were collected and processed for Co-IP assays. Co-IP was performed with rabbit anti-VCP or mouse anti-Flag antibodies, and the whole-cell lysates were analyzed for VCP, NS proteins, and β-actin expression. (**B**) PK-15 cells were transfected with pEGFP-VCP for 6 h, subsequently infected with CSFV (MOI = 1) for different time points, and stained with rabbit anti-Core antibodies (red) for confocal microscopy. (**C**) PK-15 cells were transfected with increasing amounts of VCP expression plasmid (0.5, 1, and 2 µg) or an empty vector (2 µg) for 6 h, subsequently infected with CSFV (MOI = 1) for 24 h. The expressions of VCP and Core were detected by western blotting. (**D**) PK-15 cells pre-transfected with siRNA targeting VCP were further infected with CSFV (MOI = 1) for 24 h. Cells were then harvested, and the protein expressions were analyzed by western blotting. (**E**) PK-15 cells co-transfected with pFlag-Core and pEGFP-VCP were harvested at different time points and analyzed by western blotting as described above. (**F**) PK-15 cells transfected with siVCP or siCtrl were transfected with pFlag-Core and harvested at different time intervals for western blotting analysis. (**G**) PK-15 cells were transfected with pEGFP-VCP for 6 h and then infected with CSFV (MOI = 1) for 24 h, subsequently treated with cycloheximide (CHX, 10 µg/mL) for the indicated times. Cells were then harvested, and the protein expressions were analyzed by western blotting. (**H**) PK-15 cells were transfected with siVCP or siCtrl for 24 h and then infected with CSFV (MOI = 1) for 24 h, subsequently treated with cycloheximide (CHX, 10 µg/mL) for the indicated time. Cells were then harvested, and the protein expressions were analyzed by western blotting.

Our previous study has demonstrated that VCP plays a crucial role in the replication of CSFV; however, the association between VCP and CSFV viral proteins remains insufficiently investigated. Based on this knowledge gap, the present study aimed to explore the relationship between VCP and CSFV viral proteins. Specifically, PK-15 cells were transfected with a series of CSFV protein-expressing plasmids and subsequently performed co-immunoprecipitation (Co-IP) assays. The results confirmed a specific interaction between VCP and the CSFV Core protein (Fig. 1A). Additionally, confocal microscopy was used to examine this interaction. The results showed that at 12 hours post-infection (hpi), there was no observable co-localization between Core and VCP (Fig. 1B). At 24 and 48 hpi, a discrete population of VCP co-localized with Core in infected cells, reflecting its targeted recruitment to Core-containing micro-environments, whereas the remaining VCP pool exhibited widespread cytoplasmic distribution (Fig. 1B). Next, the impact of VCP on Core stability was evaluated, and VCP overexpression resulted in a reduction in Core protein, with the Core protein inversely correlating with cellular VCP expression (Fig. 1C). In contrast, siRNA-mediated knockdown of VCP caused an increase in Core protein (Fig. 1D). These findings demonstrate an inverse relationship between the Core protein and VCP expression. Additionally, cells co-transfected with plasmids expressing Core and VCP were collected at specific time points, and western blotting data showed that VCP overexpression accelerated Core degradation (Fig. 1E), whereas the knockdown of VCP delayed the degradation process compared with the siCtrl (Fig. 1F). To further elucidate the critical role of VCP in the degradation of the CSFV Core protein, cycloheximide (CHX) chase experiments were conducted. PK-15 cells were transfected with pEGFP-VCP and subsequently infected with CSFV for 24 h. The cells were then treated with CHX for different time points to inhibit protein synthesis. These results revealed that VCP expression shortened the half-life of the Core protein, compared with that in cells transfected with the empty vector (Fig. 1G). In contrast, knockdown of VCP extended the half-life of the Core protein (Fig. 1H), indicating that VCP accelerated Core degradation. Collectively, these findings demonstrate that VCP directly interacts with the CSFV Core protein and facilitates its degradation.

### Core is degraded by VCP via the proteasome pathway

**Fig 2:**
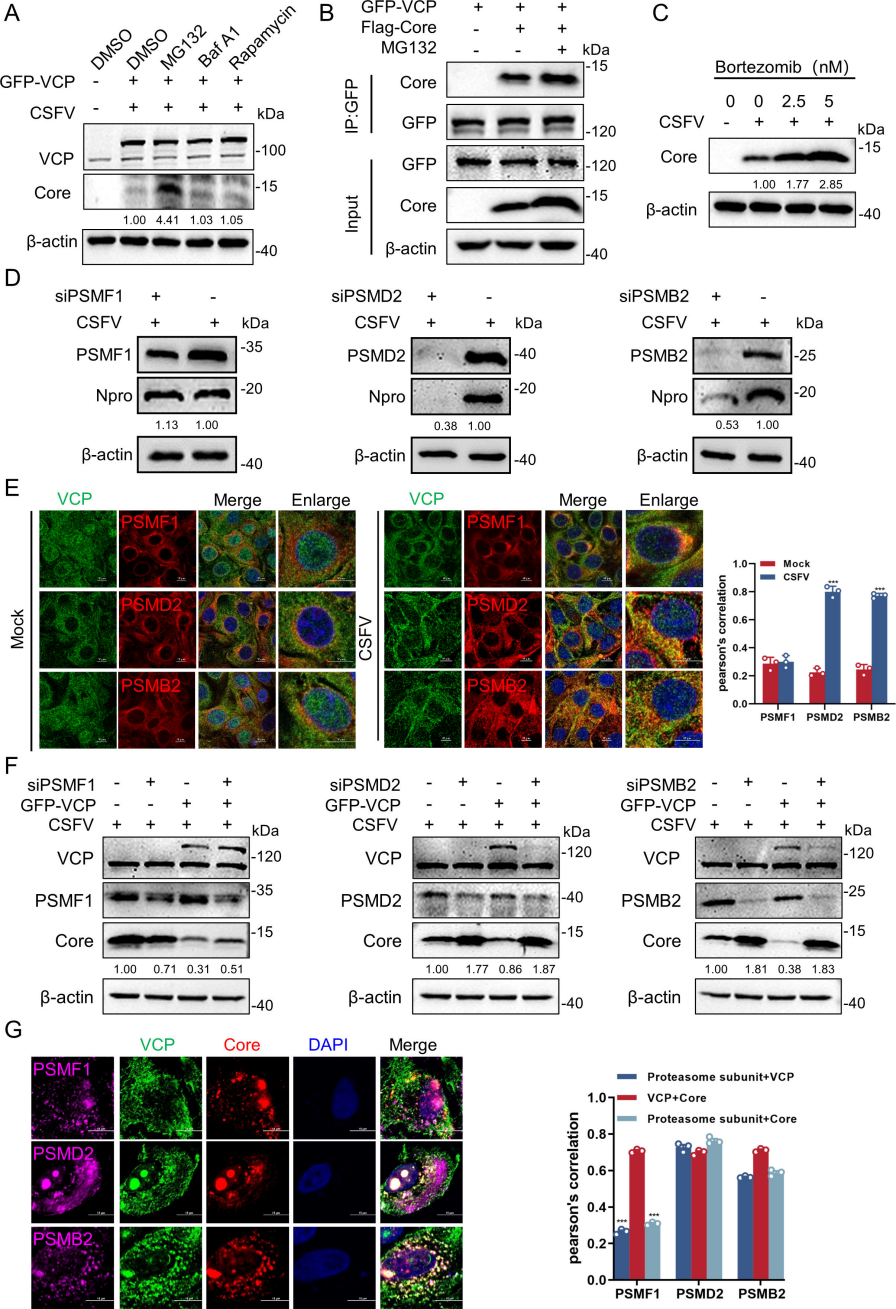
Core is degraded by VCP via the proteasome pathway. (**A**) PK-15 cells transfected with pEGFP-VCP and infected with CSFV (MOI = 1) were treated with MG132 (10 µM), rapamycin (100 nM), or BafA1 (0.4 µM) for 6 h, along with DMSO as a negative control. At 24 hpi, the cells were lysed, and Core expression was detected using western blotting. (**B**) PK-15 cells transfected with pEGFP-VCP and pFlag-Core were pre-treated with MG132 (10 µM) for 6 h and subjected to Co-IP assay using anti-GFP antibody. Proteins were immunoprecipitated with rabbit anti-GFP or a mouse anti-Flag antibody. The whole-cell lysates were evaluated for the expression of GFP-VCP, Flag-Core, and β-actin. (**C**) PK-15 cells pre-treated with concentrations of Bortezomib (0, 2.5, and 5 nM) for 6 h were infected with CSFV (MOI = 1) for 24 h and then harvested for western blotting. (**D**) siPSMF1, siPSMD2, siPSMB2, or siCtrl-transfected PK-15 cells were infected with CSFV (MOI = 1) and harvested at 24 hpi for western blotting as described above. (**E**) PK-15 cells infected with CSFV (MOI = 10) for 24 h were fixed and stained with mouse anti-VCP (green), rabbit anti-PSMF1, anti-PSMD2, or anti-PSMB2 (red), and then observed by confocal microscopy. Scale bars = 10 µm. The co-localization analysis was expressed as Pearson’s correlation coefficient. Data are means ± SD from three independent experiments. ***, *P* < 0.001. (**F**) PK-15 cells pre-transfected with siRNA-PSMF1, PSMD2, and PSMB2 were transfected with pGFP-VCP and then infected with CSFV for 24 h, and then harvested for western blotting as described above. (**G**) PK-15 cells transfected with pFlag-Core were fixed and stained with rabbit anti-PSMF1, anti-PSMD2 or anti-PSMB2 antibody (purple), goat anti-Flag antibody (red), and mouse anti-VCP (green) and observed under confocal microscopy. Scale bars = 10 µm. The co-localization analysis was expressed as Pearson’s correlation coefficient. ***, *P* < 0.001.

The proteasomal and autophagy-lysosome pathways represent the two principal mechanisms of intracellular protein degradation in eukaryotic cells. To identify the primary pathway through which VCP facilitates degradation of the CSFV Core, PK-15 cells were transfected with plasmids encoding VCP and subsequently infected with CSFV for 24 h. The cells were then treated with the proteasome inhibitor MG132, the autophagy activator rapamycin, or the autophagy inhibitor bafilomycin A1 (BafA1). Western blotting analysis revealed that treatment with MG132 effectively reversed the VCP-mediated degradation of the Core protein, whereas neither rapamycin nor BafA1 had a comparable effect (Fig. 2A). Notably, MG132 treatment enhanced the interaction between VCP and Core (Fig. 2B). This observation was further confirmed using Bortezomib—another proteasome inhibitor, which also blocked Core degradation (Fig. 2C)—confirming that Core degradation occurred in a proteasome-dependent manner. These findings strongly suggest that Core degradation is predominantly dependent on the proteasome pathway. To further validate the involvement of the proteasome in Core degradation, siRNAs targeting three proteasome subunits (PSMF1, PSMD2, and PSMB2) were employed to evaluate their roles in this process. Western blotting analysis demonstrated that knockdown of PSMD2 and PSMB2 significantly suppressed CSFV replication (Fig. 2D). Furthermore, infected cells were fixed and subjected to confocal microscopy using specific antibodies. As shown in Fig. 2E, VCP co-localized with PSMD2 and PSMB2 after CSFV infection, with Pearson’s correlation coefficient analysis supporting these data. Subsequently, the roles of PSMF1, PSMD2, and PSMB2 in VCP-mediated Core degradation were investigated. PK-15 cells with PSMF1, PSMD2, or PSMB2 knockdown were transfected with plasmids expressing VCP or an empty vector and infected with CSFV for 24 h. The results indicated that knockdown of PSMD2 and PSMB2 reversed Core degradation compared with control cells transfected with siCtrl, whereas PSMF1 knockdown had no effect (Fig. 2F), indicating that Core degradation was specifically mediated by the PSMD2 and PSMB2 subunits. Finally, the cells transfected with pFlag-Core were fixed and stained with the indicated antibodies against PSMF1, PSMD2, and PSMB2 for confocal microscopy. The results showed co-localization of PSMD2 or PSMB2 with VCP and Core (Fig. 2G), whereas PSMF1 did not co-localize (Fig. 2G), as further confirmed by Pearson’s correlation coefficient analysis. Collectively, these findings suggest that VCP facilitates Core degradation by recruiting PSMD2 and PSMB2 to form a functional proteasome complex.

### SUMO1 interacts with Core and is essential for the degradation of Core

**Fig 3:**
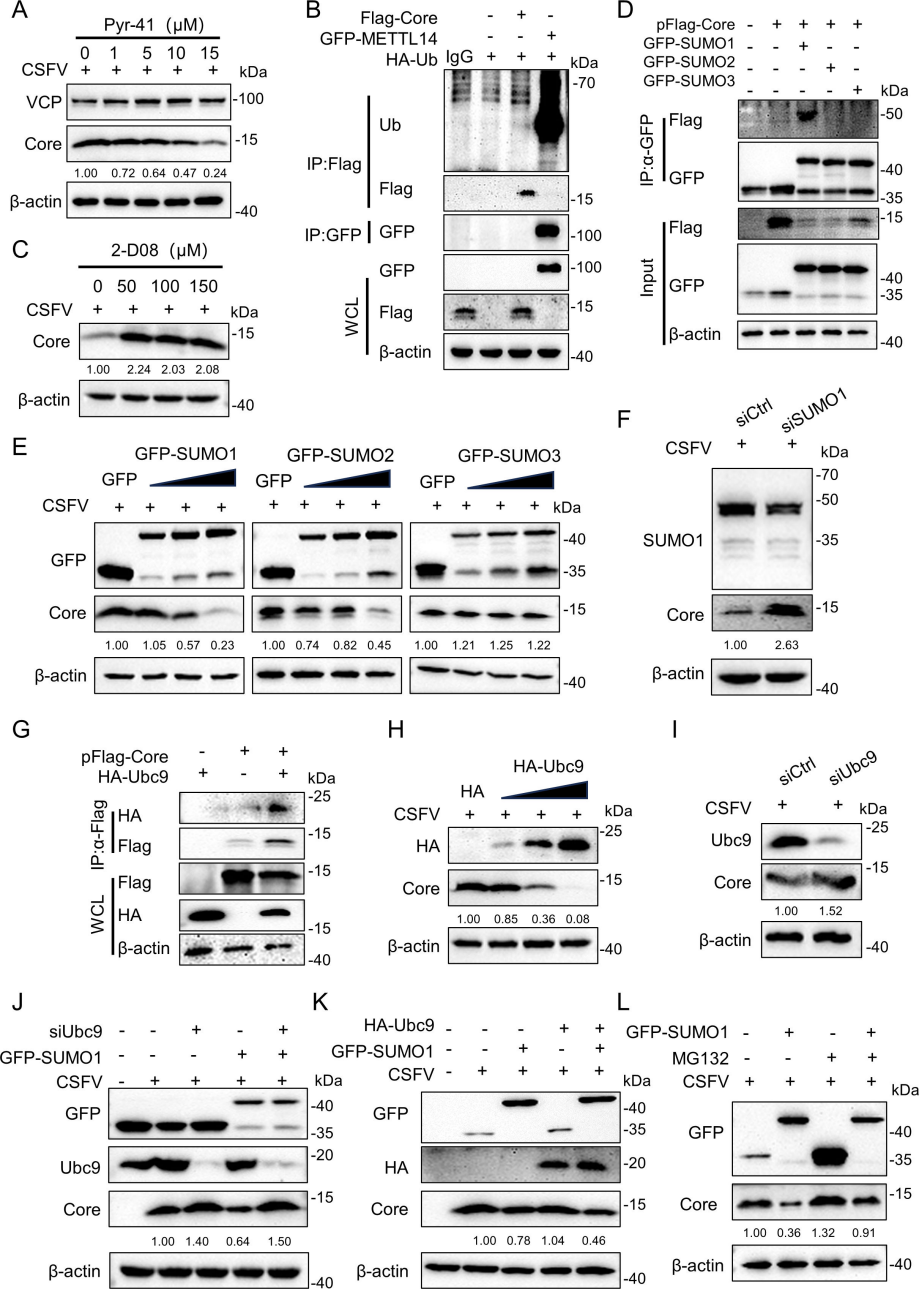
Core degradation in a SUMOylation-dependent manner. (**A**) PK-15 cells pre-treated with different concentrations of Pyr-41 (0, 1, 5, 10, and 15 nM) for 6 h were infected with CSFV (MOI = 1) for 24 h, and then, the cells were harvested at the indicated time points for protein analysis via western blotting. (**B**) HEK-293T cells co-transfected with pFlag-Core, pEGFP-METTL14, and pHA-Ub were harvested and subjected to Co-IP assay. Both cell lysates and immunoprecipitated proteins were analyzed by western blotting using mouse anti-Flag, rabbit anti-HA, and anti-GFP antibodies. (**C**) PK-15 cells were pre-treated with different concentrations of 2-D08 (50, 100, and 150 µM) for 6 h, followed by infected with CSFV (MOI = 1) for 24 h. Cells were then harvested and subjected to western blotting. (**D**) HEK-293T cells were co-transfected with plasmids expressing Flag-Core and either pEGFP-SUMO1, pEGFP-SUMO2, or pEGFP-SUMO3 for 24 h. Cell lysates were immunoprecipitated with rabbit anti-GFP antibody and analyzed by western blotting. (**E**) PK-15 cells were transfected with increasing amounts of pEGFP-SUMO1, pEGFP-SUMO2, pEGFP-SUMO3 (0.5, 1, and 2 µg) for 6 h, and then infected with CSFV (MOI = 1) for 24 h. Protein expressions of Core and SUMO1, SUMO2, SUMO3 were assessed by western blotting. (**F**) PK-15 cells pre-transfected with siRNA-SUMO1 were subsequently infected with CSFV (MOI = 1) for 24 h, and protein expressions were analyzed by western blotting. (**G**) HEK-293T cells were co-transfected with plasmids expressing pFlag-Core and pHA-Ubc9 for 24 h. Immunoprecipitation with mouse anti-Flag antibody was performed, followed by western blotting analysis. (**H**) PK-15 cells were transfected with increasing amounts of pHA-Ubc9 (0.5, 1, and 2 µg) for 6 h and then infected with CSFV (MOI = 1) for 24 h. Protein expressions of Core and Ubc9 were measured by western blotting. (**I**) PK-15 cells pre-transfected with siRNA-Ubc9 were subsequently infected with CSFV for 24 h, and protein expressions were determined by western blotting. (**J**) PK-15 cells pre-transfected with siRNA-Ubc9 were transfected with pEGFP-SUMO1 and then infected with CSFV. The cells were harvested at 24 hpi for protein analysis by western blotting. (**K**) PK-15 cells were co-transfected with pHA-Ubc9, pEGFP-SUMO1, and then infected with CSFV (MOI = 1) for 24 h. Protein expressions of Core, SUMO1, and Ubc9 were assessed via western blotting. (**L**) PK-15 cells were pre-treated with MG132 for 6 h and then transfected with GFP-SUMO1, subsequently infected with CSFV (MOI = 1) for 24 h. Protein expressions of Core and SUMO1 were assessed using western blotting.

Proteasome-mediated degradation is traditionally associated with ubiquitination (44). To determine whether ubiquitination contributes to the degradation of the CSFV Core protein, PK-15 cells were exposed to increasing concentrations of the ubiquitination inhibitor Pyr-41. Core protein expressions were subsequently assessed by western blotting analysis. Notably, inhibition of ubiquitination by Pyr-41 did not prevent Core protein degradation (Fig. 3A). To investigate the ubiquitination status of Core, HEK-293T cells were co-transfected with pFlag-Core and pHA-ubiquitin for 24 h. METTL14, a known ubiquitination substrate (45), served as a positive control. Co-immunoprecipitation (Co-IP) assays revealed robust ubiquitination of METTL14, whereas no ubiquitin signal was detected for Flag-Core, the empty vector, or IgG controls (Fig. 3B). These results indicate that Core evades ubiquitin conjugation, reinforcing the conclusion that its proteasomal degradation proceeds via a ubiquitin-independent pathway. Given that SUMOylation, a small ubiquitin-like modification, is implicated in protein degradation (46), and a previous study has highlighted a potential interaction between Core and SUMO1 (14), we hypothesized that SUMOylation would be involved in Core degradation. To investigate this, cells were treated with the SUMOylation inhibitor 2-D08, which impaired Core degradation even at a relatively low dose (Fig. 3C). In Fig. 3D, Co-IP of Flag-Core followed by immunoblotting with anti-Flag antibody revealed a prominent band with a higher molecular weight compared with the unmodified Flag-Core. This higher-molecular-weight band was consistent with the expected size of Flag-Core conjugated to SUMO (SUMOylated Flag-Core), confirming that VCP specifically interacted with the SUMOylated form of Core protein rather than its unmodified counterpart. Additionally, overexpression of SUMO1 and SUMO2, but not SUMO3, differentially suppressed Core protein expression, with SUMO1 exhibiting the strongest effect and SUMO2 showing a more modest reduction, revealing a clear gradient of efficacy among these SUMO paralogs (Fig. 3E). Conversely, siRNA-mediated knockdown of SUMO1 led to the stabilization of Core protein compared to the siCtrl group (Fig. 3F). More importantly, we also explored the involvement of Ubc9, the E2-conjugating enzyme in the SUMOylation pathway and confirmed that Core interacted with Ubc9 (Fig. 3G), with an inverse correlation between Ubc9 expression and Core protein (Fig. 3H). Knockdown of Ubc9 increased the abundance of Core protein expressions (Fig. 3I). Moreover, knockdown of Ubc9 effectively blocked the degradation Core caused by overexpression of SUMO1 (Fig. 3J), and co-transfection of SUMO1 and Ubc9 demonstrated a synergistic effect on Core degradation (Fig. 3K). Collectively, these findings suggest that SUMOylation, orchestrated by SUMO1 and Ubc9, plays a pivotal role in regulating the degradation of the CSFV Core protein. To further assess whether SUMOylation contributes to the proteasomal degradation of the CSFV Core protein, PK-15 cells were pretreated with the proteasome inhibitor MG132, followed by transfection with a pEGFP-SUMO1 overexpression plasmid and infected with CSFV. Cell lysates were harvested at 24 hpi and analyzed by western blotting. Notably, MG132 treatment abrogated the SUMO1-induced degradation of the Core protein (Fig. 3L), indicating that SUMOylation facilitates Core degradation through the proteasomal pathway. Collectively, these findings demonstrate that the CSFV Core protein undergoes proteasome-mediated degradation through a ubiquitin-independent but SUMO1/Ubc9-dependent SUMOylation mechanism.

### VCP-mediated degradation requires Core SUMOylation

**Fig 4:**
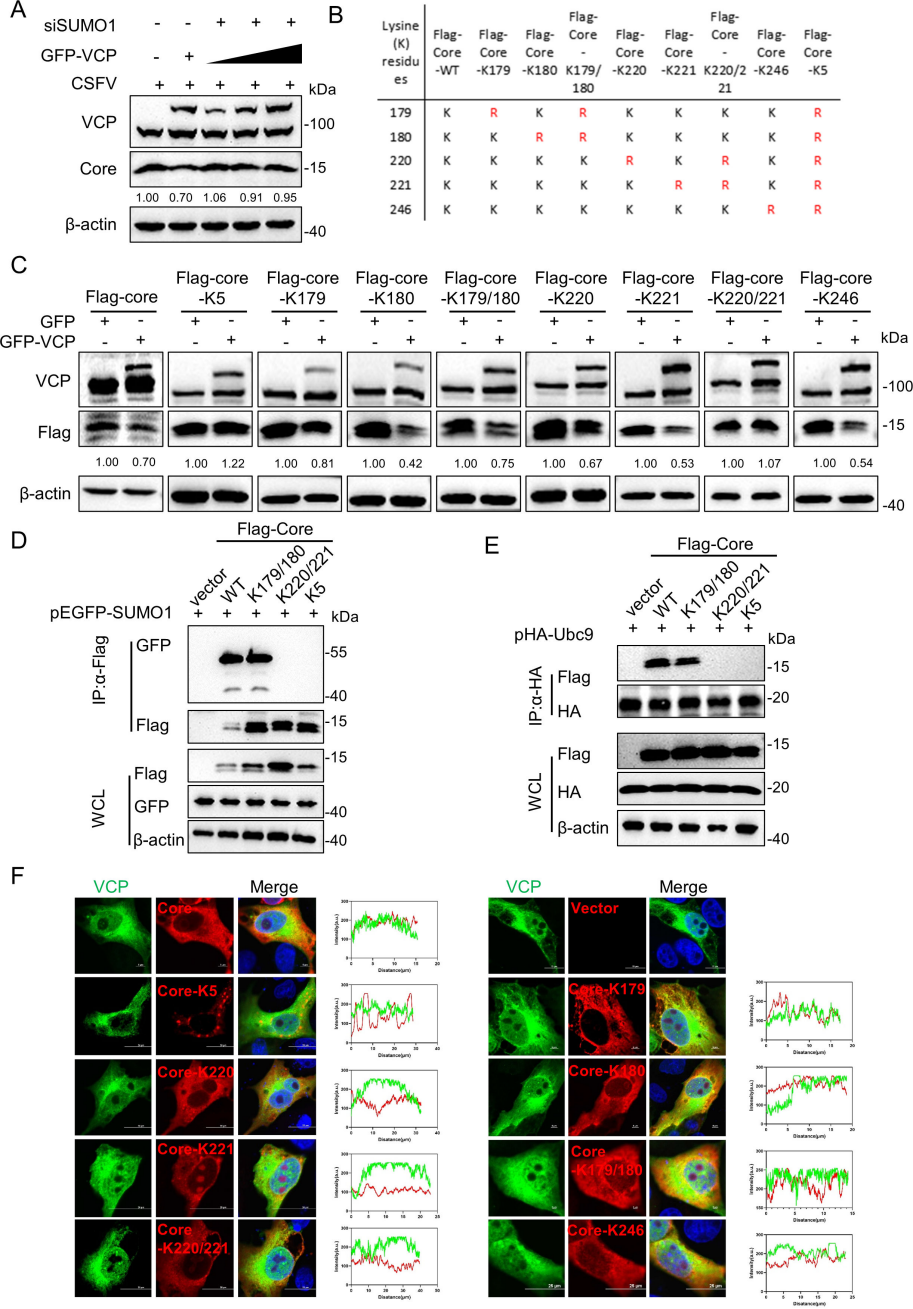
K220/221 residues on CSFV Core are critical for VCP-mediated degradation. (**A**) PK-15 cells were transfected with siRNA targeting SUMO1 for 24 h, followed by transfection with increasing amounts of pEGFP-VCP (0.5, 1, and 2 µg) and then infected with CSFV (MOI = 1). Core protein expressions were analyzed via western blotting. (**B**) A schematic diagram illustrating the SUMOylation mutants utilized in this study. (**C**) PK-15 cells were co-transfected with CSFV Core mutants and pEGFP-VCP for 24 h, and the protein expressions were assessed by western blotting. (**D**) Cells were co-transfected with pEGFP-SUMO1 and Flag-tagged core mutant proteins or Flag-empty vector (control). IP was performed with mouse anti-Flag antibody, followed by western blotting. (**E**) Cells were co-transfected with pHA-Ubc9 and Flag-tagged core mutant proteins or Flag-empty vector (control). IP was performed with rabbit anti-HA antibody, followed by western blotting. (**F**) PK-15 cells were co-transfected with pEGFP-VCP and either wild-type pFlag-Core or specific Core mutants (K5, K179, K180, K179/180, K220, K221, K220/221, and K246), with an empty vector serving as a mock control. Cells were then fixed and stained with appropriate antibodies, and the fluorescence intensity of pEGFP-VCP (green) and Core mutants (red) was quantified using the ImageJ plugin. The results were visualized and plotted using GraphPad Prism 9.

To ascertain the relationship between SUMOylation-mediated degradation of the Core protein and VCP, as well as to elucidate the underlying molecular mechanism, we first silenced SUMO1 expression in PK-15 cells using siRNA, followed by transfection with a pEGFP-VCP plasmid and subsequent CSFV infection. As anticipated, knockdown of SUMO1 attenuated Core degradation induced by VCP overexpression (Fig. 4A), suggesting that VCP facilitated Core degradation in a SUMOylation-dependent manner. Previous studies have identified amino acid residues K179, K180, and K221 of the CSFV Core protein as mediators of interaction with SUMO1, whereas residue K220 mediates interaction with Ubc9 (14). Therefore, lysine residues at positions 179, 180, 220, 221, and 246 were individually or collectively substituted with arginine (Fig. 4B). The stability of these Core mutants was assessed under VCP overexpression conditions, revealing that only mutants with all five sites altered, as well as the K220/221 group, remained resistant to degradation (Fig. 4C). This indicates that lysine residues K220/221 are critical for VCP-mediated recognition and degradation of Core. We next investigated whether K220/221 served as SUMOylation sites of Core. The results showed that after mutating K220/221 and K5 sites, Core no longer interacted with SUMO1, whereas the wild-type (WT) Core and Core with K179/180 mutation still retained the interaction with SUMO1 (Fig. 4D). Similarly, Core with K220/221 and K5 mutations also lost the interaction with Ubc9 (Fig. 4E). These findings suggest that K220/221 are SUMOylation sites of Core. Furthermore, confocal microscopy was employed to assess the co-localization of VCP with the Core mutants. In alignment with our hypothesis, VCP failed to co-localize with the Core-K5 and K220/221 mutants (Fig. 4F), whereas the Core-WT and other mutants maintained co-localization with VCP (Fig. 4F), reinforcing the notion that K220/221 were essential for VCP-mediated degradation. Collectively, these findings demonstrate that SUMOylation of Core at residues K220/221 is crucial for its recognition and subsequent degradation mediated by VCP.

### VCP facilitates CSFV uncoating by transporting virus to the late endosome

**Fig 5:**
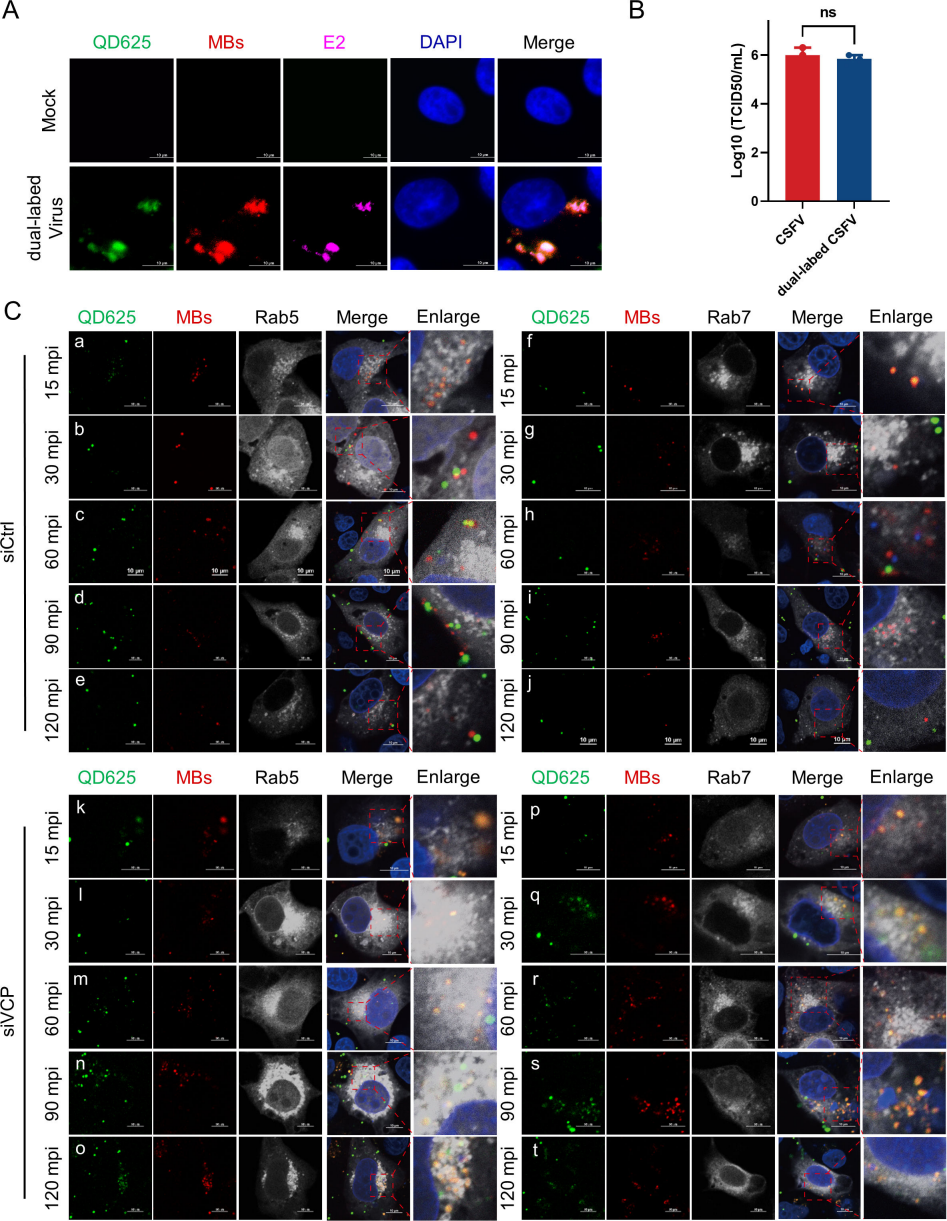
VCP facilitates CSFV transport to the late endosome for uncoating. (**A**) PK-15 cells were infected with dual-labeled CSFV (100 µL) for 4 h. Cells were fixed and stained with mouse anti-E2 (purple), and the nucleus was stained with DAPI (blue). The images were captured via confocal microscopy. (**B**) Titers of CSFV and dual-labeled CSFV replication in PK-15 cells. (**C**) PK-15 cells infected by dual-labeled CSFV at different time points post-infection (*n* = 50). Scale bar = 10 µm. Magnified images of dotted boxes are shown in the merged images.

The results presented above have demonstrated that VCP interacts with the CSFV Core protein and mediates its degradation, and the CSFV Core protein serves as the nucleocapsid protein of this virus. This observation has further extended our interest in investigating whether VCP is involved in the uncoating of CSFV. Building upon established JEV genome tracking methodologies. The envelopes of purified CSFV were labeled with streptavidin-modified quantum dots (SA-QDs) by intercalating biotinylated lipids into the lipid layer. In addition, a multivalent fluorescence amplification strategy was used as a facile and competent tool for highly sensitive RNA imaging to label the RNA genome while maintaining the integrity of the viral RNA (47). To determine whether the dual-labeled viruses specifically labeled CSFV, we first evaluated their co-localization with the viral E2 protein using an immunofluorescence assay (IFA). Confocal microscopy revealed that nearly all QD625 and MMBs signals co-localized with E2 signals, whereas negligible signals were observed in the negative control group (Fig. 5A), confirming the specificity of the dual-labeling strategy. We further assessed the impact of dual labeling on viral infectivity. Compared with unlabeled viruses, labeling CSFV with QD625 and MMBs had a minimal effect on its infectivity (Fig. 5B). This efficient dual-labeling approach enabled us to monitor the release behavior of individual RNA genomes at the single-virus level. To observe viral uncoating, dual-labeled viruses were first incubated with PK-15 cells at 4°C for 30 min to allow membrane binding without endocytosis. Subsequently, the temperature was raised to 37°C to initiate infection, and the cells were fixed at different time points. After 15 min, dual-labeled viruses accumulated in the perinuclear region of cells, and yellow signals—representing the overlap of envelope (QD625) and RNA genome (MMBs)—were partially co-localized with Rab5, whereas some remained dispersed in the cytoplasm (Fig. 5C-a). At this stage, minimal yellow fluorescence was observed in Rab7-positive compartments (Fig. 5C-f). By 30 min post-infection (mpi), most yellow signals were localized within Rab5-positive endosomes (Fig. 5C-b), whereas separated MMBs signals started to appear in Rab7 compartments (Fig. 5C-g), suggesting the onset of viral uncoating. At 60 mpi, only a few yellow signals remained in Rab5 compartments. Instead, QD625 and MMBs signals were clearly separated and no longer co-localized with Rab5 (Fig. 5C-c). In contrast, distinct QD625 and MMBs signals appeared within Rab7-positive endosomes (Fig. 5C-h), indicating that extensive uncoating of CSFV had occurred. By 90 mpi, yellow signals were nearly absent from Rab5 compartments (Fig. 5C-d and e), whereas numerous dissociated QD625 and MMBs signals accumulated in Rab7 compartments (Fig. 5C-i and j), marking the completion of genome release. However, upon VCP knockdown, a large number of virus particles remained trapped within Rab5-positive compartments (Fig. 5C-k through o), consistent with our previous observations (23). Notably, strong yellow fluorescence signals were retained within Rab5-positive, but not Rab7-positive compartments (Fig. 5C-p through t), indicating that genome-envelope separation was inhibited. These findings suggest that CSFV uncoating occurs in the late endosomal stage, where the viral RNA genome separates from the envelope. VCP facilitates this uncoating process by promoting endosomal maturation; its depletion restricts virus particles to early endosomes, thereby impairing CSFV uncoating and subsequent infection.

### K220/221 is essential for CSFV transport to the late endosome for uncoating

**Fig 6:**
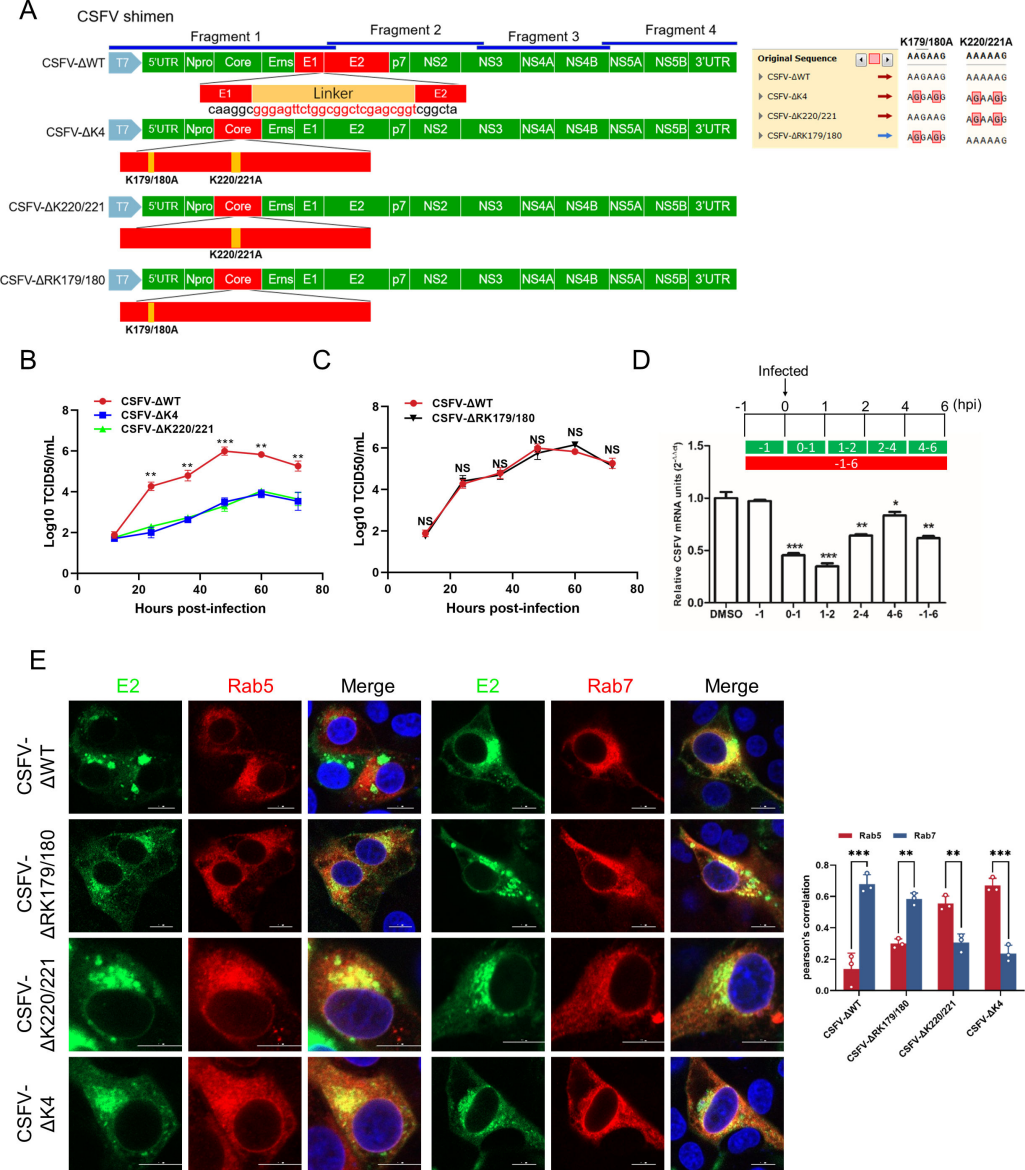
K220/221 is essential for CSFV proliferation and transport. (**A**) Schematic representation of the CSFV wild-type and mutants used in this section. (**B and C**) PK-15 cells were infected with the parental and recombinant CSFV. The intracellular CSFV titers were determined at the indicated time points by IFA. (**D**) PK-15 cells were treated with MG132 and then infected with CSFV, following the schematic, the CSFV mRNA was measured by RT-qPCR. (**E**) PK-15 cells were infected with wild-type and mutant viruses for 24 h, and the cells were fixed and stained with mouse anti-E2 antibody (green) and rabbit anti-Rab5 or Rab7 antibody (red). The images were captured using confocal microscopy.

To probe the functional significance of these residues in viral replication, K-to-R substitutions were introduced into an infectious full-length cDNA clone of the highly virulent CSFV strain Shimen. The rescued mutant viruses, named CSFV-ΔK220/221 and CSFV-ΔK4, contain a combination of K-to-R substitutions at residues K179, K180, K220, and K221 in the Core protein (Fig. 6A). PK-15 cells were subsequently infected with recombinant viruses harboring site-directed mutations in the Core protein: CSFV-ΔWT, CSFV-ΔK4, or CSFV-ΔK220/221 (MOI = 1). Viral titers were measured at the indicated time points. Notably, mutant viruses CSFV-ΔK4 and CSFV-ΔK220/221 exhibited significantly reduced titers compared with the parental CSFV-ΔWT virus (Fig. 6B), indicating impaired viral replication. Furthermore, to assess whether restoration of SUMOylation sites could rescue viral fitness, a compensatory mutation was introduced into the CSFV-ΔK4 background, generating the CSFV-ΔRK179/180 revertant virus (Fig. 6A). However, the replication capacity of this revertant remained similar to that of CSFV-ΔWT (Fig. 6C), demonstrating that restoration of K220/221 alone is sufficient to fully recover viral replication. PK-15 cells were treated with the proteasome inhibitor MG132 and subsequently infected with CSFV as outlined in Fig. 6D. Administration of MG132 during the first 0–1 hpi and 1–2 hpi markedly suppressed viral replication (Fig. 6D), indicating that proteasomal activity played a pivotal role in the CSFV uncoating process. In parallel, confocal microscopy was employed to assess whether a CSFV Core mutant affects viral trafficking from Rab5-positive early endosomes to Rab7-positive late endosomes. The analysis demonstrated that substitution of the K220/221 residues significantly impaired the translocation of CSFV from Rab5 to Rab7 compartments (Fig. 6E). These findings establish that the SUMOylation sites within Core are critical determinants of CSFV’s endosomal trafficking from early to late compartments, thereby facilitating efficient uncoating. In summary, our findings delineate the critical role of lysine residues K220/221 in the degradation of the Core protein, mediated through a SUMOylation-dependent mechanism. Furthermore, the disruption of these residues impairs viral propagation, highlighting their indispensable role in the life cycle of CSFV.

### Core degradation requires the VCP-NPL4-UFD1 complex

**Fig 7:**
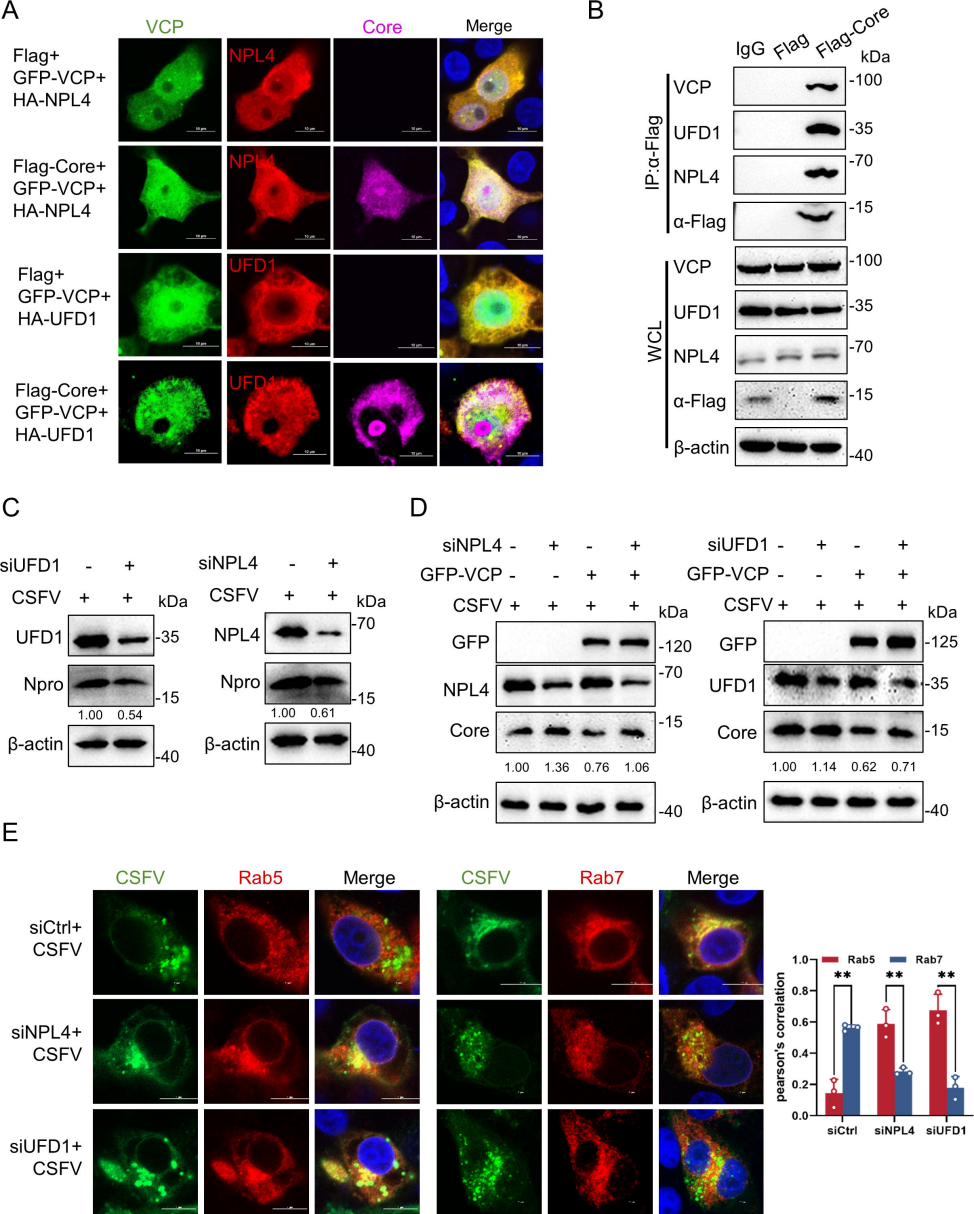
UFD1 and NPL4 act as the cofactor of VCP. (**A**) PK-15 cells co-transfected with pFlag-Core, pEGFP-VCP, and either pHA-NPL4 or pHA-UFD1 were fixed and stained with rabbit anti-HA antibody (red) and mouse anti-Flag antibody (purple), followed by confocal microscopy observation. Scale bars = 10 µm. (**B**) HEK-293T cells were transfected with pFlag-Core for 24 h. Cell lysates were immunoprecipitated using anti-Flag antibody, and the resulting proteins were analyzed by western blotting with specific antibodies. (**C**) PK-15 cells transfected with siRNA targeting NPL4 or UFD1 were infected with CSFV (MOI = 1) and harvested at 24 hpi for protein analysis via western blotting. (**D**) PK-15 cells pre-transfected with siRNA targeting NPL4 or UFD1 were transfected with pEGFP-VCP and then infected with CSFV (MOI = 1) for 24 h. Cells were then harvested, and protein expressions were analyzed by western blotting as described above. (**E**) PK-15 cells pre-transfected with siRNA targeting NPL4 or UFD1 were infected with CSFV for 24 h, and the cells were fixed and stained with mouse anti-E2 antibody (green) and rabbit anti-Rab5 and Rab7 antibodies (red), followed by confocal microscopy observation. Scale bars = 10 µm.

VCP exhibits limited intrinsic substrate specificity on its own and relies on various cofactors to execute its diverse functions (48). To identify the potential cofactors of VCP involved in Core degradation, we employed confocal microscopy to assess the interactions between VCP, Core, and NPL4 or UFD1. In cells transfected with an empty vector, VCP co-localized with both NPL4 and UFD1 (Fig. 7A). In cells transfected with pFlag-Core, both NPL4 and UFD1 co-localized with VCP and Core (Fig. 7A). To further validate these interactions, the Co-IP assay was performed. Cells were transfected with pFlag-Core for 24 h and subjected to Co-IP assay using a Flag monoclonal antibody. The results confirmed that Core interacted with VCP, UFD1, and NPL4 (Fig. 7B). The results above indicate that VCP forms a complex with NPL4 and UFD1 to bind Core. Furthermore, siRNAs targeting NPL4 or UFD1 were transfected into PK-15 cells, which were then infected with CSFV. At 24 hpi, the cells were harvested and analyzed by western blotting. The results revealed a significant decrease in CSFV Npro protein expression in both NPL4- and UFD1-knockdown cells (Fig. 7C), suggesting that NPL4 and UFD1 were involved in regulating CSFV replication. To investigate whether NPL4 and UFD1 participate in the VCP-mediated degradation of Core, we observed that knockdown of NPL4 or UFD1 impeded VCP-mediated Core protein degradation (Fig. 7D). These results confirm that VCP forms a trimeric complex with NPL4 and UFD1 to mediate the degradation of the Core protein. Furthermore, we also investigated the role of UFD1 and NPL4 in the trafficking process of CSFV. The results showed that knockdown of UFD1 and NPL4 impaired the transport of CSFV from Rab5 to Rab7 (Fig. 7E).

## DISCUSSION

**Fig 8:**
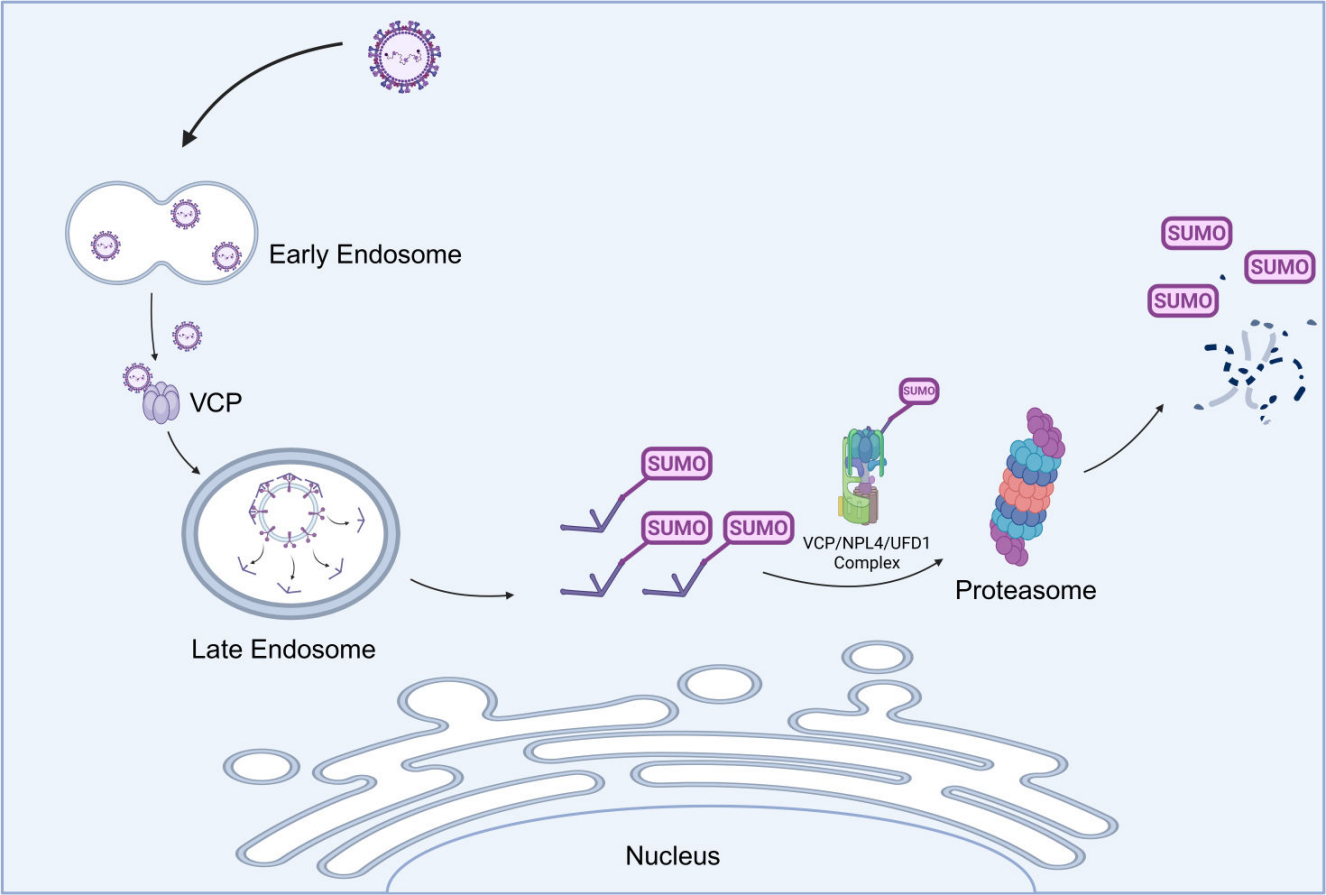
Model of SUMOylation-dependent Degradation of Nucleocapsid in CSFV uncoating. After CSFV virions enter cells, internalized virions are transported to the late endosome for uncoating. CSFV Core protein undergoes SUMOylation in a Ubc9, SUMO1-dependent manner, with VCP interacting with the SUMOylated Core to mediate its subsequent degradation. Mechanistically, VCP plays a pivotal role in the translocation of Core from the early to late endosome, facilitated by the co-factors NPL4 and UFD1. The SUMOylated Core is then degraded through the ubiquitin-independent PSMB2 and PSMD2 26S proteasome system(created with BioRender.com).

VCP is a multifunctional ATPase involved in a variety of cellular processes, regulating protein homeostasis, and has been implicated in the life cycle of multiple viruses (38). In the *Flaviviridae* family, particularly the *Flavivirus* genus, VCP plays a pivotal role during early infection stages. It supports the replication cycle of flaviviruses, including ZIKV (26), YFV (24), WNV (29), and DENV (25), facilitating key processes such as viral uncoating, replication organelle formation, and the disassembly of stress granules. Building on our previous work that demonstrated the importance of VCP in CSFV infection through pharmacological inhibition, overexpression, and RNA interference (23). Our current findings highlight VCP’s essential role in Core protein degradation and viral uncoating (Fig. 8). First, we demonstrate that VCP physically interacts with the CSFV Core protein, and modulating the VCP expression alters Core protein expression. Second, VCP coordinates endosomal trafficking: VCP knockdown impairs CSFV transit from early to late endosomes, blocking uncoating. Collectively, these data indicate that VCP facilitates viral uncoating by promoting Core degradation, establishing a functional link between VCP-mediated disassembly of viral components and the progression of productive CSFV infection.

Proteasomal degradation is typically initiated via ubiquitination. However, CSFV Core degradation deviates from this paradigm: Core lacks ubiquitination, and its stability is unaffected by the ubiquitin-activating enzyme inhibitor Pyr-41-consistent with previous reports (49). Instead, our results substantiated that the non-ubiquitinated Core engaged the SUMOylation pathway, interacting with SUMO1 and Ubc9, which aligns with findings in macrophages (14). SUMOylation is increasingly recognized as a mechanism hijacked by viruses to regulate host-pathogen interactions and promote replication (50). Our study identified SUMOylation as a critical mediator of CSFV Core uncoating and degradation—a distinct mechanism for *Flaviviridae*. Specifically, we demonstrated that Core SUMOylation was a prerequisite for its recognition and processing by VCP and the proteasome. RNAi-mediated knockdown of SUMO1 or Ubc9 confirmed that Core degradation depends on SUMOylation, suggesting that this modification may represent a broader viral strategy to bypass the canonical ubiquitin–proteasome pathway. We further mapped the key residues driving Core SUMOylation: lysines K220 and K221 were indispensable for SUMO1/Ubc9-mediated modification, which in turn enabled recognition and degradation by VCP. This aligns with prior reports that SUMOylation acts as a proteasomal degradation signal in a ubiquitin-independent manner, particularly in the context of viral protein quality control. Critically, mutating K220/221 not only stabilized Core but also severely impaired CSFV replication, underscoring these residues’ essential role in the viral life cycle. Notably, compensatory restoration of K220/221 failed to rescue replication, suggesting that SUMOylation requires context-dependent (and potentially sequential) modification, involving cooperative action of multiple lysine residues. These results highlight a previously unappreciated mechanism by which host SUMOylation machinery and VCP coordinate to control CSFV protein turnover and infectivity, offering potential targets for antiviral intervention.

Previous reverse genetics studies have demonstrated that although the CSFV Core protein is not strictly required for *in vitro* virion assembly, it is pivotal for efficient replication and *in vivo* pathogenicity. For instance, deleting most of the Core gene (VP447ΔC) severely impairs viral proliferation, which can be partially rescued by a compensatory N2177Y point mutation in the NS3 protein (51). Although this mutation restored particle production *in vitro*, the resulting virus was completely attenuated in pigs, suggesting that NS3 partially substitute Core in virion assembly, but not in mediating full infectivity or pathogenicity. Recent work further underscores that genomic RNA harboring NS3 compensatory mutations can be packaged without Core, emphasizing the cis-acting role of NS3 in genome encapsidation (52). These findings align with those of our current study, in which we demonstrate that Core not only contributes to viral infection but also undergoes VCP- and SUMOylation-dependent degradation during uncoating, a process critical for efficient CSFV infection. Although NS3 may structurally compensate for Core in certain contexts, the regulated degradation of Core is likely required for proper uncoating and early infection events—functions that NS3 cannot fully replicate. Together, these observations underscore the multifaceted role of the Core protein in both structural integrity and dynamic post-entry processes essential for viral propagation.

VCP activity is primarily modulated through its interaction with distinct cofactors. We identified that UFD1 and NPL4 bind to VCP, forming a complex involved in the degradation and transport of Core. Knockdown of VCP, UFD1, or NPL4 inhibited CSFV replication. Notably, UFD1 or NPL4 knockdown prevented VCP-induced Core degradation. These findings align with previous reports showing that VCP plays a role in the degradation of other viral proteins, such as the coronavirus nucleocapsid N protein (34) and the ZIKV capsid protein through a ubiquitin-dependent pathway (26).

Despite defining the role of SUMOylation in Core degradation during uncoating, our study has several limitations. First, the lack of suitable Core-specific antibodies prevented direct evaluation of how SUMOylation alters the RNA-binding affinity of Core; instead, we relied on indirect readouts such as viral replication, protein stability, and mutant phenotypes. Second, although the K220/221 mutations likely disrupt SUMOylation, potential impacts on RNA binding cannot be excluded. To address this, we examined mutations at a distinct lysine site (K179/180), which had no impacts on replication, supporting the specificity of the K220/221 phenotype. Finally, SUMOylation appears to function post-entry, as modifications observed during ectopic expression may not reflect physiological regulation during assembly. Clarifying the timing and dynamics of Core SUMOylation will be an important direction for future work.

In conclusion, our study is the first to demonstrate that SUMOylation of the Core protein plays a pivotal role in CSFV uncoating. We propose a model where CSFV utilizes VCP to extract SUMOylated Core, recruit proteasome subunits to form a proteolytic complex, and release its genome following uncoating (Fig. 8). These findings not only deepen our understanding of the CSFV life cycle but also reveal potential therapeutic targets for antiviral strategies. Targeting the VCP-SUMOylation pathway could offer a novel approach for inhibiting CSFV replication, contributing to the control and eradication of CSF. Future studies should explore the broader implications of SUMOylation in viral uncoating and evaluate its potential as a therapeutic target across various viral families.

## MATERIALS AND METHODS

### Virus, cell line, drugs, and antibodies

CSFV Shimen strain (Genbank access number AF092448) used in this study was described previously (23). Porcine kidney (PK-15) cells were cultured in Dulbecco’s modified Eagle’s medium (DMEM; GIBCO) supplemented with 10% fetal bovine serum (FBS) (GIBCO, Invitrogen), 100 mg/mL streptomycin, and 100 IU/mL penicillin (GIBCO, Invitrogen), and maintained at 37°C in a humidified atmosphere with 5% CO_2_. The compounds MG132, Rapamycin, BafA1, Bortezomib, Pyr-41, and 2-D08 were purchased from commercial vendors (MCE, USA). All the antibodies utilized in this study were procured from commercial sources, as detailed in Table S1.

### Plasmids and transfection

The plasmids pFlag-E2, -Core, -NS3, -NS4B, NS5A, -NS5B, pHA-Ub, and pEGFP-VCP used in this study were stored in our laboratory. Ubc9, SUMO1, SUMO2, and SUMO3 cDNAs were obtained by RT-PCR from total RNA extracted from PK-15 cells. The cDNAs corresponding to Ubc9, SUMO1, SUMO2, and SUMO3 were amplified by PCR and cloned into vectors to generate pHA-Ubc9, pEGFP-SUMO1, pEGFP-SUMO2, and pEGFP-SUMO3 plasmids. The authenticity of all constructs was verified by DNA sequencing. Core SUMOylation mutants (K179, K180, K220, K221, K246, K179/180, K220/221, and K5) were generated by site-directed mutagenesis (C214, Vazyme) from pFlag-Core. PK-15 cells grown to 70% confluence on coverslip dishes were transfected with the plasmid using Lipofectamine 3000 (Invitrogen) according to the manufacturer’s instructions. At 6 h post-transfection (hpt), the transfection mixture was replaced with DMEM containing 2% FBS, and the cells were incubated for an additional 24 h. For RNA knockdown, cells were transfected with siRNA using Lipofectamine RNAiMAX (Invitrogen) according to the manufacturer’s instructions. The sequences of siRNAs targeting VCP, PSMF1, PSMB2, PSMD2, SUMO1, and Ubc9 were synthesized from Sangon Biotech and are listed in Table S2. The siRNAs targeting UFD1 (sc-41689) and NPL4 (sc-61227) were purchased from Santa Cruz.

### Virus labeling

In this experiment, the external envelope and internal viral genome of the CSFV were simultaneously labeled with quantum dots and MMBs as Liu et.al describe (47). The specific procedures were as follows: 0.1 mg DSPE-PEG (2000)-Biotin (Avanti) was carefully weighed to react with 100 µL of purified viruses (the viral concentration was 1 mg/mL) at 37°C for 2 h. The viruses were then mixed with 1 µL MMBs (100 µM, Sangon, Shanghai), the sequences of MMBs are listed in Table S3, and oscillated and shielded from light at 37°C for 2 h. NAP-5 desalting column (GE Healthcare) was then used to remove excess, unbound DSPE-PEG (2000)-Biotin and the MBs. Subsequently, the PK-15 cells were incubated with the viruses at 4°C for 30 min and washed three times with a cold PBS buffer. SA-QDs were added to the cell-culture dish at 4°C for 30 min, and the cells in the Petri dishes (NEST, China) were washed three times before being photographed. Before the experiment, the viruses and SA-QDs were filtered using a 0.22 µm filter to eliminate aggregates.

### Confocal microscopy

PK-15 cells were grown in 35 mm Petri dishes (NEST, GBD-35-20) with a glass bottom. After treatment, the monolayers were fixed with 4% PFA in PBS and permeabilized with 0.1% Triton X-100. To visualize the CSFV structural and Core proteins, the cells were stained with mouse anti-Flag antibody. For visualization of proteasome subunit and VCP, the cells were stained with mouse anti-VCP antibody, or rabbit anti-PSMF1, PSMD2, or PSMB2 antibody. To visualize the co-localization of VCP, Core, UFD1, or NPL4, the cells were stained with mouse anti-Flag and rabbit anti-UFD1 or NPL4. Images were acquired using a Nikon A1 laser scanning confocal microscope equipped with a 60 × oil immersion objective. Sequential scanning was performed to prevent spectral overlap. Imaging parameters—including laser intensity, exposure time, and detector gain—were carefully optimized to prevent signal saturation and ensure accurate fluorescence representation. All images within the same experiment were captured under identical settings. Post-acquisition processing was limited to linear adjustments of brightness and contrast using Nikon NIS-Elements software. Co-localization was quantitatively assessed using the Nikon A1 software, and the results were expressed as Pearson’s correlation coefficients. Co-localization analysis was performed using NIS-Elements software (Nikon Instruments, Japan). For quantitative evaluation, regions of interest (ROIs) were delineated around areas exhibiting co-localized signals from the two fluorophores, and the Pearson’s correlation coefficient was calculated across all pixels within these ROIs. Values of PCC were obtained from at least three independent experiments, and the representative data are presented.

### Coimmunoprecipitation assay and western blotting

Cells co-transfected with pEGFP-VCP and pFlag-E2, -Core, -NS3, -NS4B, -NS5A, or -NS5B, were lysed in NP-40 lysis buffer. A 25% aliquot of the supernatant (whole cell lysate, WCL) was removed from all samples for later use. The remaining 75% of the lysates were treated with mouse anti-Flag antibody or rabbit anti-VCP antibody and incubated with rotation for 6 h at 4°C, and then, 40 µL of a protein A/G Plus-agarose slurry (sc-2003; Santa Cruz) was added to the lysate for 4 h at 4°C with rotation. The agarose beads were washed with NP-40 by centrifugation at 1,000 × *g* for 5 min at 4°C four times. The agarose beads were collected by centrifugation and resuspended in 2 × SDS loading buffer for SDS-PAGE and western blotting. The cell samples were washed three times with ice-cold PBS and then lysed in RIPA lysis buffer (R0020, Solarbio) supplemented with protease inhibitors (Sigma) for 30 min at 4°C. Lysates were clarified by centrifugation at 12,000 *× g* for 10 min at 4°C. A 120 µL aliquot of the supernatant was removed from all samples for later use, then resuspended in a 5 × SDS loading buffer. Protein samples were separated by SDS-PAGE and transferred to nitrocellulose membranes. After blocking with 5% skim milk for 1 h at 37°C, the membranes were incubated with primary antibodies overnight at 4°C, followed by incubation with corresponding horseradish peroxidase-conjugated secondary antibodies for 1 h at 37°C. The immunolabeled protein complexes were visualized using the ECL Plus kit (Jacob enzyme Biotech, SQ201) and using the Tanon 5200 scanner system (Tanon, China). β-actin was used as a loading control. To determine the expression of indicated proteins, the corresponding protein/actin quantity was used to calculate the grayscale values using ImageJ 7.0 software.

### Construction of CSFV Core mutants

The CSFV mutants were generated using the T7 *in vitro* transcription system. The complete genome of the CSFV Shimen strain was divided into four overlapping PCR fragments, with each fragment containing more than 200 bp of overlap with adjacent fragments to ensure seamless coverage of the viral genome. A T7 promoter sequence was incorporated into the forward primer of the first fragment. These fragments were amplified via PCR (NEB, Ipswich, MA) and cloned into the low-copy-number plasmid pWSK29. The full-length infectious clone was assembled through fusion PCR and designated as CSFV-ΔWT. Based on the CSFV-ΔWT backbone, two mutant constructs, CSFV-ΔK4 and CSFV-ΔK220/221, were developed by substituting lysine (K) residues with arginine (R) through site-directed mutagenesis. These mutations were introduced using the Mut Express II Fast Mutagenesis Kit (Vazyme, China), following the manufacturer’s instructions. The CSFV-ΔRK179/180 mutant was generated based on CSFV-ΔK4 by substituting the arginine residues at positions 220 and 221 with lysine. The assembled construct served as a template for *in vitro* transcription. The ligated cDNA was used to synthesize RNA with a T7 transcription kit (Promega, USA). Full-length genomic RNA, along with RNA from the mutant constructs, was transfected into PK-15 cells using the DMRIE-C transfection reagent (GIBCO, Invitrogen). After a 24 h recovery period in growth medium, the cells were washed with PBS twice and cultured in the maintenance medium for 3 days.

### Statistical analysis

All data were presented as means ± standard deviations (SD) as indicated. Student’s *t*-test was used to compare the data from pairs of treated and untreated groups. Statistical significance is indicated by asterisks in the figures. All statistical analyses and calculations were performed using Prism 9 (GraphPad Software, Inc., La Jolla, CA)

## Data Availability

All relevant data are within the article.

## SUPPLEMENTAL MATERIAL

Supplemental tables jvi.01648-25-s0001.docx Tables S1 to S3.

ASM does not own the copyrights to Supplemental Material that may be linked to, or accessed through, an article. The authors have granted ASM a non-exclusive, world-wide license to publish the Supplemental Material files. Please contact the corresponding author directly for reuse.
