# Supplementary material for: SUMOylation-dependent degradation of nucleocapsid is responsible for Pestivirus uncoating
Source: J Virol. 2025 Nov 25;99(12):e01648-25. doi: 10.1128/jvi.01648-25 (PMC12724189; doi:10.1128/jvi.01648-25)
Supplement: Supplemental tables — Tables S1 to S3. [file jvi.01648-25-s0001.docx]

Table S1 Antibodies used in this study.

| Antibody | Name | Supplier | Catalog no. |
| --- | --- | --- | --- |
| VCP(R) | VCP Rabbit Polyclonal antibody | Proteintech | 10736-1-AP |
| Lamp-1 | Lamp1 Rabbit Polyclonal antibody | Proteintech | 55273-1-AP |
| NPL4 | NPLOC4 Polyclonal antibody | Proteintech | 11638-1-AP |
| UFD1 | UFD1L Polyclonal antibody | Proteintech | 10615-1-AP |
| GFP | GFP tag polyclonal Ab | Proteintech | 50430-2-AP |
| GAPDH | GAPDH Mouse Monoclonal antibody | Proteintech | 60004-1-Ig |
| Calnexin | Rabbit polyclonal to Calnexin | abcam | ab22595 |
| β-actin | β-Actin Rabbit mAb (High Dilution) | Abclonal | AC026 |
| PSMF1 | PSMF1 Antibody | CUSABIO | CSB-PA852882DSR1HU |
| PSMB2 | PSMB2 Antibody | CUSABIO | CSB-PA01385A0Rb |
| PSMD2 | PSMD2 Antibody | CUSABIO | CSB-PA619755LA01HU |
| Flag | Anti-Flag M2 antibody | Sigma-Aldrich | F1804 |
| HA | Anti-HA Antibody Mouse MAb | Sigma-Aldrich | H3663 |
| CSFV E2 | Mouse Monoclonal Anti-CSFV E2 antibody | A gift from Prof Qin Wang (National Institute of Veterinary Drug Control, Beijing, China)). | |
| CSFV Core | Rabbit polyclonal Anti-CSFV Core antibody | A gift from Dr.Lei Feng ( Jiangsu Academy of Agricultural  Sciences, Nanjing, China) | |
| CSFV Npro | Mouse monoclonal Anti-CSFV Npro antibody | A gift of Prof Zhiyong Ma (Shanghai Veterinary Research Institute, Chinese Academy of Agricultural Sciences, Shanghai, China). | |

Table S2 siRNA duplexes in this study.

| Primer | Sequence (5’-3’) | Acession  Number | Use |
| --- | --- | --- | --- |
| siNC | AATTCTCCGAACGTGTCACGT | AJ312193 | Control interference RNA |
| siVCP | GAAUAGAGUUGUUCGGAAUTT | NM214280.1 | VCP interference RNA |
| siPSMF1 | CCUGUAUGUCCUCCGGUAU | NM001243340.1 | PSMF1 interference RNA |
| siPSMD2 | GCGCCAGTTAGCTCAATAT | NM001243477.1 | PSMD2 interference RNA |
| siPSMB2 | GGAGCUUCUUAGGAAAUGUTT | XM021095920.1 | PSMB2 interference RNA |
| siSUMO1 | AGGAAGAAGAUGUGAUUGATT | NM001112676.1 | SUMO1 interference RNA |
| siUbc9 | GCCAAGAAGUUUGCUCCCUTT | NM001204369.1 | Ubc9 interference RNA |

Table S3 MB sequence in this study.

| MB  name | Sequence (5’-3’) |
| --- | --- |
| MB-1 | /i2OMeC//i2OMeG//i2OMeC//i2OMeA//i2OMeC//i2OMeC//i2OMeA/*/i2OMeA/*/i2OMeC/*/i2OMeA/*  /i2OMeC/*/i2OMeA/*/i2OMeU/*/i2OMeA/*/i2OMeA/*/i2OMeC/*/i2OMeA/*/i2OMeC/*/i2OMeC/*/i2OMeU/  */i2OMeA//i2OMeG//i2OMeG//i2OMeU//i2OMeG//i2OMeC//i2OMeG/ |
| MB-2 | /i2OMeC//i2OMeG//i2OMeC//i2OMeA//i2OMeC//i2OMeC//i2OMeA/*/i2OMeA/*/i2OMeA/*/i2OMeU/*  /i2OMeA/*/i2OMeU/*/i2OMeC/*/i2OMeA/*/i2OMeA/*/i2OMeG/*/i2OMeU/*/i2OMeA/*/i2OMeC/*/i2OMeA/*  /i2OMeA//i2OMeG//i2OMeG//i2OMeU//i2OMeG//i2OMeC//i2OMeG/ |

“i2OMe” represents 2’-O-methyl RNA modification and “*” represents phosphorothioate modification.
